# From Social to Financial: Understanding Trust in Extended Payment Services on Social Networking Platforms

**DOI:** 10.3390/bs15050659

**Published:** 2025-05-12

**Authors:** Qian Zhang, Heejin Kim

**Affiliations:** College of Business Administration, Gachon University, Seongnamdaero, Sujeong-gu, Seongnam-si 13120, Gyeonggi-do, Republic of Korea; zq0924@gachon.ac.kr

**Keywords:** social networking services (SNSs), mobile payment, hedonic motivation, utilitarian motivation, cognitive trust, emotional trust, behavioral intention, schema congruity theory

## Abstract

Considering the rapid increase in mobile payment usage, numerous big tech companies have added mobile payment to the primary services that their platforms offer. However, extant research predominantly treats this added service as a standalone offering and investigates user adoption and behavior for this service independent of the primary services. Recognizing this gap in the literature, this study considers the added service as part of an extended ecosystem and examines different motivations for using the primary service. Therefore, this study examines how different motivations for using social networking services (SNSs) shape trust in the extended payment service and ultimately influence behavioral intentions. Drawing on the schema congruity theory, we conceptualize trust as a multidimensional construct—distinguished between cognitive and emotional trust—and explore the impact of trust in the primary service on the use of an added service. Specifically, we analyze survey data of 478 users of South Korea’s leading SNS. The results reveal that both hedonic and utilitarian motivations positively influence emotional and cognitive trust, which, in turn, drive behavioral intention. However, hedonic (utilitarian) motivation exerts a stronger effect on emotional (cognitive) trust. Overall, the findings enhance the knowledge regarding trust formation in extended service ecosystems and offer insights for tech firms integrating financial services into their platforms.

## 1. Introduction

Mobile payment is a payment method that utilizes a mobile device to make purchases or transfer money (J. Lee et al., 2019). Mobile payments have become a dominant global payment method, with the penetration rate in the United States reaching 89% in 2022 (Anan et al., 2022). The total worldwide transaction value was USD 9.46 trillion in 2023 and is projected to grow to USD 14.78 trillion by 2027 (Yadav et al., 2024). This growth is driven by advancements in financial services and information and communication technology (ICT; J. Lee et al., 2019; Tang et al., 2021). Initially led by financial institutions, such as banks and credit card companies, large technology companies have increasingly entered this market through “fintech” strategies (Ng et al., 2022).

Among technology platforms, social networking services (SNSs) have emerged as a prominent context for embedded payment solutions (J. Lee et al., 2019). Unlike fintech firms that center their offerings around financial services, SNS platforms integrate payment functionalities as complementary extensions of their core services to enhance user satisfaction (J. Lee et al., 2019). For example, Tencent’s WeChat Pay seamlessly embeds payment functionality within its social messaging app (X. Li et al., 2023), and Facebook Pay facilitates transactions within its social networking ecosystem (Newaz et al., 2023).

SNS platforms offer utilitarian and hedonic benefits (Akdim et al., 2022; Anderson et al., 2014; Xu et al., 2012). They serve practical functions, such as maintaining connections and accessing information (Syn & Oh, 2015) while providing entertainment and allowing for creative expression and social engagement (Mensah et al., 2018). The primary value that each user derives from SNSs varies, and the motivations and benefits perceived by the user shape their experiences and influence their perceptions of extended services.

Regarding the extension to payment services, trust is an important factor that shapes users’ adoption of such services. As SNS users increasingly conduct transactions and share personal information, concerns over privacy breaches, data misuse, and hacking have grown (Gefen & Straub, 2004). Moreover, mobile payments are considered higher risk owing to their financial implications and digital vulnerabilities (Jebarajakirthy & Shankar, 2021; J. Lin et al., 2014; Pal et al., 2021; Zhou, 2011a). Such users rely on psychological reassurance to overcome perceived threats, which helps ease concerns regarding digital transaction environments (Oliveira et al., 2016; Suh & Han, 2002). Therefore, trust is needed to ease such concerns and to facilitate mobile adoption within SNSs.

Traditionally, trust has been conceptualized as unidimensional (Alalwan et al., 2015; de Blanes Sebastián et al., 2023; Kilani et al., 2023); however, recent studies highlight its multidimensional nature (Calefato et al., 2015; Johnson & Grayson, 2005; Leong et al., 2021; Wang et al., 2023). A widely utilized framework distinguishes between cognitive trust (rational confidence in reliability) and emotional trust (affective attachment and security; Leong et al., 2021; Wang et al., 2023). As cognitive trust and emotional trust develop through distinct mechanisms, understanding both dimensions is essential for designing effective marketing strategies (Wang et al., 2023).

We propose that user motivations for using primary services influence the formation of different types of trust. Specifically, hedonic (utilitarian) motivations align with (foster) emotional (cognitive) trust. In extended payment services, these trust mechanisms shape consumer adoption behaviors. Research links motivations to trust and service adoption in the areas of internet banking (Alalwan et al., 2015), online payments (de Blanes Sebastián et al., 2023), e-wallets (Kilani et al., 2023), and mobile payments (Zhou, 2014b). Nevertheless, most studies treat trust as a unidimensional construct, overlooking its multifaceted nature, particularly in technology-based ecosystems where hedonic and utilitarian motivations coexist (Nan et al., 2020). Additionally, they primarily examine services as standalone offerings and not how perceptions of a primary service influence the adoption of extended services. This is particularly important in SNS-based payment services, where existing user engagement and platform familiarity may shape trust-building processes differently from those in traditional financial services. Our study bridges these gaps by investigating how hedonic and utilitarian motivations influence emotional and cognitive trust in mobile payments. Applying the theories of schema congruity and spillover effect, we explore how these trust mechanisms shape behavioral intentions toward extended payment services.

We surveyed users of Kakao, a dominant tech company in South Korea. KakaoTalk (a mobile SNS) commands a 99.4% usage rate among mobile messengers in South Korea (Y. W. Park & Lee, 2019). Its extended service, KakaoPay, had 40 million users in 2023. This case provides an ideal setting to analyze how user motivations for a primary service influence trust in its extended payment service.

The remainder of this study is structured as follows: Section 2 presents the theories of schema congruity and spillover effect, our research framework’s foundation, and a literature review on hedonic and utilitarian motivations and their relationship with trust, as well as cognitive and emotional trust. Subsequently, we present a conceptual model outlining our hypotheses and explain how trust is formed and transferred within integrated social and financial platforms. Section 3 describes the methodology. Section 4 elaborates on the results. Finally, Section 5 presents this study’s theoretical contributions and managerial implications, future research directions, and our conclusions.

## 2. Literature Review and Hypothesis Development

### 2.1. Theoretical Framework

#### 2.1.1. KakaoPay: Transforming Mobile SNSs to Mobile Payment Services

KakaoPay—launched in 2014 on the KakaoTalk platform (J. Lee et al., 2019), a leading South Korean SNS—is the country’s most popular mobile payment platform (Nan et al., 2020; Zhao & Pan, 2023). The service enables users to make online and offline payments, transfer money, and manage financial transactions seamlessly through their mobile devices. As of 2023, KakaoPay had over 39.4 million registered users, substantially outpacing other mobile wallet providers such as Samsung Pay, SK Pay, L Pay, and LG Pay.

Compared with other digital payment services, KakaoPay is unique in that it was launched within a social media platform. Other similar services include WeChat Pay (X. Li et al., 2023) and Facebook Pay (Newaz et al., 2023). KakaoTalk is a social messaging platform launched in 2010 and has 43 million active users in a country of 51.6 million people (B. Kim, 2024). The integration of KakaoPay within KakaoTalk has several implications. First, KakaoTalk provides an extensive user base for KakaoPay (Y. W. Ha et al., 2015; Nan et al., 2020). Second, this integration creates a comprehensive ecosystem wherein users directly access financial services within the SNS (Nan et al., 2020). As such, users’ perceptions and experiences of the two services are interconnected (X. Li et al., 2023).

However, few studies have examined user behavior in the situations described above. Previous studies have primarily focused on service adoption and usage and applied the technology adoption model (Nan et al., 2020) and unified theory of acceptance and use of technology (de Sena Abrahão et al., 2016; X. Lin et al., 2021) to provide insights into the characteristics of SNS-based payment services. However, scholars have typically employed frameworks designed for traditional payment services and overlooked the primary social media platforms’ unique influence on their integrated payment service. These limitations highlight the need to investigate how users’ experiences with an SNS shape their behavior toward related payment services within the same platform ecosystem.

#### 2.1.2. Spillover Effect

Spillover theory explains how experiences in one domain influence perceptions and behaviors in another (Shan et al., 2024; Zhang et al., 2019), frequently through schema-based processing (Simonin & Ruth, 1998). For instance, the positive evaluation of a similar product influences that of a new product in the same or related category (Carter & Curry, 2011). This effect is well documented in the context of brand extension (Aaker & Keller, 1990; Bitner, 1990). In financial services, research on the transfer of trust has demonstrated a spillover effect—from online to offline environments (Teng et al., 2023) and web-based to mobile platforms (Zhang et al., 2019). Gefen and Straub (2004) found that trust developed in e-commerce settings enhances consumer confidence in offline transactions with the same company. Likewise, trust in traditional online banking services positively influences the adoption of mobile banking solutions (G. Kim et al., 2009).

Both cognitive knowledge and emotions are transferable between entities. Functional attributes, such as reliability and ease of use (Aaker & Keller, 1990), as well as positive emotions associated with a parent brand, such as satisfaction and joy (Bitner, 1990), have been demonstrated to transfer to new products, thus enhancing their acceptance and perceived value. In sum, spillover effects operate in cognitive and emotional domains, influencing rational evaluations and emotional connections.

#### 2.1.3. Schema Congruity Theory

The schema congruity theory posits that individuals tend to maintain cognitive consistency with pre-existing schemas (Meyers-Levy & Tybout, 1989). This theory is widely utilized in consumer research, including advertising claims (Cheong & Kim, 2011), brand image evaluation (Bhaduri, 2019), and brand engagement (Gerrath & Biraglia, 2021). According to this theory, when individuals encounter information consistent with their activated schema, such information is processed more easily and receives greater attention owing to cognitive ease (Meyers-Levy & Tybout, 1989). This processing ease makes individuals more likely to generate positive evaluations (Zhang et al., 2019). These cognitive dynamics are central to how preferences, trust, and attitudes are formed, especially when quick judgments are necessary (Bhaduri, 2019; Bhaduri et al., 2017).

Several factors influence which pre-existing schema is activated and how consistency with new information is determined. First, goals are among the most influential factors as they guide which schemas are brought to the forefront during information processing (Kruglanski et al., 2002). For example, according to Peng et al. (2023), utilitarian (hedonic) goals typically activate schemas focused on practicality and functionality (pleasure and emotional satisfaction). Second, contextual cues, including the environment or framing of information, can trigger specific schemas (A. Y. Lee & Labroo, 2004). Previous experiences are also crucial in shaping and reinforcing certain schemas as they are readily accessible when similar situations arise (Gollwitzer, 1999). Moreover, an individual’s emotional state can influence which schemas are activated, with positive emotions typically activating more favorable schemas (S. S. Kim, 2009). Finally, social influences, including peer pressure and cultural norms, can direct schema activation by aligning individual perceptions with the group’s perceptions (Briley et al., 2000). These factors collectively determine the extent of schema congruity and subsequently influence consumers’ information processing, preferences, choices, and attitudes.

### 2.2. Hypothesis Development

**Figure 1:**
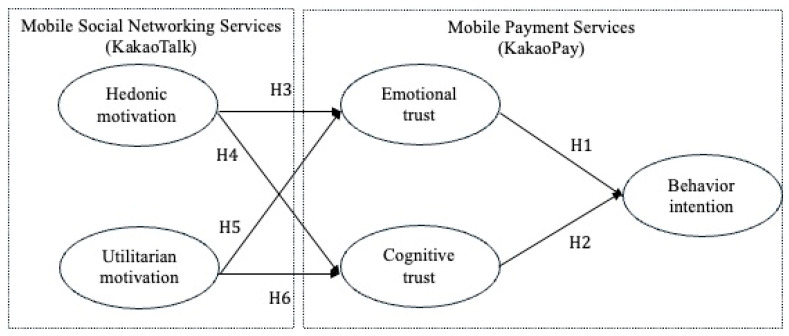
Conceptual framework of this research. Source: author’s drawing.

The following subsection describes this study’s hypotheses and conceptual framework, as illustrated in Figure 1.

#### 2.2.1. User Trust and Behavioral Intention for KakaoPay

Emotional trust differs from cognitive trust, as distinguished by Leong et al. (2021) and Wu et al. (2023). Emotional trust arises from feelings of safety and comfort with a service (Gong et al., 2020; Leong et al., 2021). It is shaped by emotional responses and personal experiences and greatly affects user behavior (Choi & Lee, 2017; Gong et al., 2020). By contrast, cognitive trust is based on rational confidence in a service’s reliability and benefits, which is formed through deliberate evaluations (Choi & Lee, 2017; Gong et al., 2020). It emerges from consumers’ reasoned assessments and evidence-based beliefs (Choi & Lee, 2017). In mobile payment services, emotional trust pertains to users’ feelings of security regarding mobile payment adoption (Gong et al., 2020). Users who feel assured and comfortable with mobile payment services are more likely to adopt them. For example, Gong et al. (2020) found that emotional trust predicted users’ intention to use a service.

Studies suggest that emotional trust primarily arises from personal experiences of interactions with a given service. Hence, the quality and quantity of interactions are key factors. For instance, the strength of relationships (Calefato et al., 2015), frequency, and duration of contact (Johnson & Grayson, 2005) greatly impact emotional trust. SNS-based mobile payment services such as KakaoPay benefit from their integration into platforms that users frequently engage with (Y. W. Ha et al., 2015). This high frequency and extended interaction foster emotional trust as users build comfort and assurance through regular use within a familiar social media context. These interactions enhance the user’s overall trust in the service, strengthening their intention to use it. Accordingly, we hypothesize the following:

**H1.**  *Emotional trust positively influences the intention to use mobile payment services.*

Cognitive trust involves users’ belief in the service’s ability to provide reliable and authentic mobile payment transactions (Gong et al., 2020). Multiple studies highlight the importance of cognitive trust in consumers’ usage behavior regarding payment services (Dimitriadis & Kyrezis, 2010; Gong et al., 2020; Leong et al., 2021), as mobile transactions are generally perceived to involve higher risks (Zhou, 2013). Moreover, users perceive major risks associated with SNS platforms owing to data privacy concerns, particularly considering past security breaches (Chan & Saqib, 2015). For example, mobile networks are vulnerable to hacker attacks, information interception (Zhou, 2014a), and information disclosure concerns (Zhou & Li, 2014). Location-based services also increase users’ privacy concerns and perceived risks (Chopdar et al., 2018; Zhou, 2011b), including the possibility of security threats and fraud (Chauhan, 2024). As such, cognitive trust’s role may be even more critical for payment services integrated into SNSs. Therefore, we hypothesize the following:

**H2.**  *Cognitive trust positively influences the intention to use mobile payment services.*

#### 2.2.2. Motivation for the Use of Primary Services and Trust in Extended Services

Motivation is critical in shaping and directing human behavior (Deci & Ryan, 1985). In SNSs, user motivation significantly influences user experiences with respective platforms (Kwak et al., 2014). Spillover theory suggests that user motivations in SNSs may influence trust in related extended services. As users engage with the primary service, their motivations shape their perceptions and experiences. This, in turn, may influence their trust in the extended service.

For instance, positive experiences with SNSs, such as satisfaction with communication or ease of use, may enhance users’ cognitive trust in an extended service by shaping their perceptions of its reliability and competence (H. Y. Ha et al., 2016). Likewise, emotional experiences with the SNS, such as enjoyment or trust in the platform, may spill over to foster emotional trust in the extended service. This conjecture is supported by research showing that trust developed in one context may be transferred to a related domain, for example, from e-commerce to offline transactions (C. C. Huang et al., 2020) or from online to mobile banking services (G. Kim et al., 2009).

Here, we examine how hedonic and utilitarian motivations influence trust in extended services. Hedonic motivation greatly enhances users’ trust in various service contexts such as web services (Hwang & Kim, 2007) and online shopping (Chang et al., 2016). For instance, Chang et al. (2016) demonstrated that increased hedonic value strengthens cognitive trust and emotional attitudes while reducing perceived risks. Thus, we hypothesize the following:

**H3.**  *Hedonic motivation to use mobile SNSs positively influences emotional trust in extended mobile payment services.*

**H4.**  *Hedonic motivation to use mobile SNSs positively influences cognitive trust in extended mobile payment services.*

Utilitarian motivations, which include communication, information gathering, and networking, may also substantially influence trust (Thomas & Jadeja, 2021; Wongkitrungrueng & Assarut, 2020). Individuals motivated by the practical benefits of using SNSs tend to prioritize attributes, such as reliability, competence, and security, which are key factors in forming cognitive trust (Gefen & Straub, 2004). When users perceive a service as effective in fulfilling their practical needs, this perception enhances cognitive trust. Consistent and efficient performance fosters emotional security, which contributes to emotional trust (Johnson & Grayson, 2005).

Regarding SNSs, research demonstrates that utilitarian attributes, such as the perceived usefulness of information gathered through social media, influence cognitive and emotional trust (H. Y. Ha et al., 2016). Likewise, within a platform environment, the utilitarian motivation to use SNSs can foster positive cognitive and emotional trust, which may extend to related services. Thus, we hypothesize the following:

**H5.**  *Utilitarian motivation to use mobile SNSs positively influences emotional trust in extended mobile payment services.*

**H6.**  *Utilitarian motivation to use mobile SNSs positively influences cognitive trust in extended mobile payment services.*

#### 2.2.3. The Strength of the Relationship Between Motivation and Trust

The schema congruity theory posits that when new stimuli align with existing schemas, they are more easily processed with less effort (S. S. Kim, 2009). If so, what schemas are accessible when evaluating new stimuli? One factor that influences which schema is activated is the individual’s goal (Carter & Curry, 2011; Zhang et al., 2019). Goals strongly shape the cognitive schemas that are activated and relied upon during information processing (Kruglanski et al., 2002). For instance, when an individual is driven by a utilitarian goal (focused on practical and functional outcomes), schemas emphasizing the cognitive and logical aspects of a given piece of information (Peng et al., 2023) are likely to be activated. Conversely, a hedonic goal (centered on pleasure and emotional gratification) activates schemas highlighting the affective and experiential aspects (Peng et al., 2023).

Accordingly, we hypothesize that the type of goal that an individual holds influences the dimension of trust that they prioritize. Specifically, individuals with hedonic goals prioritize the affective aspects of trust. Driven by the pursuit of pleasure and emotional connection, their schemas result in them assessing trust based on emotional resonance and relational warmth. Conversely, individuals with utilitarian goals primarily focus on the cognitive aspects of trust. Their activated schemas prioritize functionality and efficacy; hence, they evaluate trust based on logical assessments of the trustee’s abilities and dependability (Peng et al., 2023). Empirical evidence supports this conjecture. C. Kim et al. (2020) showed that hedonic (utilitarian) value strongly influences emotional (cognitive) trust. Therefore, we hypothesize the following:

**H7.**  *The positive effect of hedonic motivation for mobile SNSs on emotional trust in extended mobile payment services exceeds that of utilitarian motivation.*

**H8.**  *The positive effect of utilitarian motivation in mobile SNSs on cognitive trust in extended mobile payment services exceeds that of hedonic motivation.*

## 3. Research Methodology

### 3.1. Research Design

This study utilized SPSS 26.0 and AMOS 26.0 software to analyze the data. First, SPSS 26.0 was employed to conduct a reliability analysis of the survey data. This analysis, using Cronbach’s alpha, assessed the internal consistency of the scales. Subsequently, AMOS 26.0 was used to perform CFA and evaluate the model’s fit. Composite reliability (CR) and average variance extracted (AVE) were calculated to verify convergent validity. Discriminant validity was confirmed by comparing the square roots of the AVE with the correlations between constructs. To address potential common method bias, a single-factor test was conducted using AMOS. The results indicate that the single-factor model’s fit was substantially worse than that of the measurement model, suggesting that common method bias did not pose a significant concern. Finally, if a study’s goal is to test and validate theoretical frameworks, covariance-based structural equation modeling (CB-SEM) is the appropriate method. Conversely, if the focus is on prediction and theoretical development, partial least squares SEM (PLS-SEM) is more suitable (Dash & Paul, 2021). As we aimed to validate theoretical constructs, we used AMOS 26.0 to conduct the analysis and CB-SEM to evaluate the proposed hypotheses.

### 3.2. Measures

To ensure data quality and relevance, a screening question was included at the beginning of the survey to confirm that participants had prior experience using KakaoTalk. Only those who indicated that they had used KakaoTalk were allowed to proceed with the rest of the questionnaire; all others were screened out. We adopted and modified certain measures used in previous studies to fit our study context. Measures of hedonic motivations (HMs) for KakaoTalk (seven items) were obtained from Salimon et al. (2017), and those of utilitarian motivations (UM; eight items) were obtained from Chen et al. (2013) and H. Lee and Cho (2020). Measures of cognitive trust (CT) in KakaoPay (three items) were obtained from Dwyer et al. (2007) and G. Kim et al. (2009), and those of emotional trust (ET) for KakaoPay (three items) were obtained from Komiak and Benbasat (2006). Behavioral intention (BI) toward KakaoPay was measured using two items on whether users will continue to use KakaoPay in the future. In summary, 23 items extracted from the five constructs were used in the questionnaire. A seven-point Likert scale (1 = strongly disagree; 7 = strongly agree) was used (Appendix A).

### 3.3. Data Collection Process

This study was approved by the University Institutional Review Board (IRB) for Bioethics (Approval Code: 1044396-202406-HR-096-01). Before commencing the survey, respondents were informed of the research objectives, procedures, and their right to withdraw at any time. They were assured that their data would remain anonymous and confidential, after which they provided their informed consent to participate in the study. Data were collected only from those who consented, and all responses were anonymized.

The survey was administered among adults aged 20 and older using the online platform Tillian Pro (Pro.tillionpanel.com), a professional online research panel in South Korea. A stratified sampling method was employed based on age and gender to ensure a balanced distribution of respondents. Upon survey completion, participants received reward points equivalent to USD 0.40, which they could accumulate and redeem for vouchers through the panel platform.

We recruited 478 valid respondents, which exceeded the recommended minimum sample size of 200 for structural equation modeling to ensure sufficient statistical power (Dash & Paul, 2021). Stratified sampling based on age and gender was employed to enhance demographic representativeness among South Korean adults aged 20 and above.

Table 1 presents the sample’s descriptive statistics. Among the 478 respondents, 50.8% and 49.2% were male and female, respectively, with a mean age of 45.63 years.

## 4. Data Analysis and Results

### 4.1. Reliability and Validity Analysis

A reliability analysis was conducted using SPSS 26.0 to evaluate the internal consistency of the scales. All Cronbach’s alpha values exceeded 0.7, indicating high internal reliability (Table 2).

Subsequently, CFA was performed to evaluate the overall validity of the measurement model. The CFA results demonstrated an acceptable model fit (Browne & Cudeck, 1992). The absolute fit indices were χ^2^(df) = 220(0.000); χ^2^/df = 4.023; RMSEA = 0.08; IFI = 0.932; and CFI = 0.932. Convergent validity was assessed using average variance extracted (AVE) and construct reliability (CR), and their values exceeded the recommended thresholds of 0.50 and 0.70, respectively (Fornell & Larcker, 1981). Thus, the measurement model fulfilled the established validity and reliability criteria (refer to Table 2).

To assess discriminant validity, we compared the squared correlations between two distinct weights in either construct, which should be below the AVEs (Fornell & Larcker, 1981). Table 3 lists the results. All square roots of AVEs exceeded the correlation between the constructs comprising each pair.

### 4.2. Common Method Bias

To ensure anonymity, the links to the surveys on KakaoTalk and KakaoPay were sent separately. Subsequently, we verified that the data were not prone to common method bias because we loaded all items onto one common factor in a CFA framework (S. Huang et al., 2022; C. Li & Huang, 2024; Podsakoff et al., 2003; Zenker et al., 2021; χ^2^ = 2717.09; df = 230; χ^2^/df = 39.375 > 3; *p* < 0.001; CFI = 0.744; GFI = 0.573; NFI = 0.727; IFI = 0.744; RMSEA = 0.151).

### 4.3. Path Analysis and Hypothesis Testing

Table 4 presents the results. The model fit indices indicate that the structural model is adequately supported by the data, thus confirming its overall goodness of fit: χ^2^(223) = 1091; df = 4.893; *p* < 0.001; RMSEA = 0.900; CFI = 0.911; IFI = 0.911; NFI = 0.890; GFI = 0.833; and TLI = 0.899. Emotional (β = 0.575, t = 8.108, *p* < 0.001) and cognitive trust (β = 0.267, t = 3.954, *p* < 0.001) significantly influenced behavioral intention, supporting H1 and H2. Hedonic motivation significantly influenced emotional (β = 0.487; t = 5.821, *p* < 0.001) and cognitive (β = 0.466, t = 5.667, *p* < 0.001) trust, supporting H3 and H4. Likewise, utilitarian motivation significantly affected emotional (β = 0.265, t = 3.193, *p* < 0.001) and cognitive (β = 0.298, t = 3.635, *p* < 0.001) trust, supporting H5 and H6.

**Figure 2:**
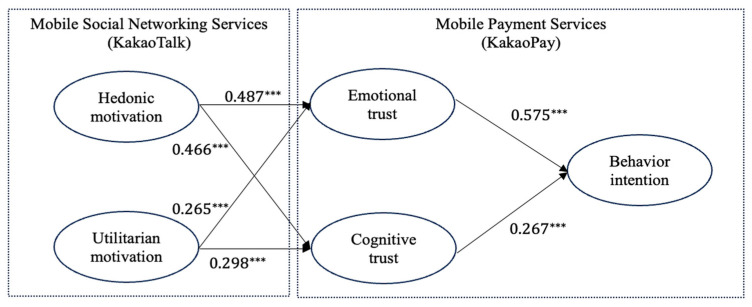
SEM results. Note: *** *p*-value < 0.001; →: significant path.

Finally, hedonic motivation more strongly affected emotional (β = 0.487, t = 5.821, *p* < 0.001) than cognitive (β = 0.466, t = 5.667, *p* < 0.001) trust, supporting H7. Conversely, utilitarian motivation more strongly affected cognitive (β = 0.298, t = 3.635, *p* < 0.001) than emotional (β = 0.265, t = 3.193, *p* < 0.001) trust, supporting H8. Figure 2 illustrates the results.

## 5. Discussion

Social exchange theory suggests that trust is a fundamental component of exchange relationships as individuals assess the risks and benefits of engaging in transactions (Cook & Emerson, 1987). This principle extends beyond interpersonal relationships to financial transactions, where trust is essential because of the presence of information asymmetry and individuals’ perceived risks and dependence on intermediaries (Gefen, 2000). Prospective users must trust the system’s security and reliability, as well as the service provider’s integrity, before adopting mobile payment services (Lian & Li, 2021).

Although extensive research has examined trust as a key determinant of financial service adoption (Chin et al., 2022; Lian & Li, 2021), few studies have explored how trust is formed and transferred across diverse service domains. This gap is particularly important in the service context of technology-based companies (J. Lee et al., 2019), which continuously expand their ecosystems by integrating diverse services. As firms increasingly embed financial solutions within non-financial platforms, such as SNSs (J. Lee et al., 2019; X. Li et al., 2023; Newaz et al., 2023), understanding when and how trust is transferred between these distinct service categories becomes crucial. While technology-driven and financial services may be inherently different, users’ trust could still be transferred between them. However, this phenomenon has hardly been explored.

We address this gap by examining how motivations for using a primary SNS influence trust formation in the extended payment service of the SNS. Our results demonstrate that consumers’ psychological inclinations toward the primary service influence their behavioral intentions toward the extended service. Specifically, the results demonstrate that hedonic motivation significantly influences emotional trust, whereas utilitarian motivation significantly influences cognitive trust. These findings support prior research (C. Kim et al., 2020) suggesting that emotional trust is formed through affective experiences and enjoyment, whereas cognitive trust is grounded in performance expectations and rational evaluations. Additionally, both emotional trust and cognitive trust positively influence behavioral intention, highlighting emotional trust’s importance in addition to cognitive factors that influence trust, such as security and transaction speed. Moreover, when the users’ motivation to use SNS is hedonically oriented, emotional trust plays a bigger role in shaping behavioral intention. By contrast, when utilitarian motivation is high, cognitive trust is more important. This finding extends schema congruity theory to the trust context and sheds light on the importance of understanding users’ motivations for using a platform in predicting their adoption of extended services.

### 5.1. Theoretical Implications

First, this study contributes to the literature on mobile payment by highlighting how the characteristics and prior experiences of a platform’s existing users shape adoption behavior. Research has primarily examined mobile payment as an independent service, focusing on general adoption and usage behavior with the assumption of a homogeneous user base (Alalwan et al., 2015; de Blanes Sebastián et al., 2023; Kilani et al., 2023; Zhou, 2014b). However, when mobile payment is introduced as an extended service by a technology firm, the primary users are those already engaged with the company’s core offerings (Y. W. Ha et al., 2015; Nan et al., 2020). Recognizing this unique situation, we examined the representative case of users who actively use KakaoTalk to analyze their behavioral intentions toward KakaoPay. We enhance the literature by highlighting how service integration within an ecosystem influences user behavior, extending beyond isolated adoption frameworks.

Second, this study contributes to brand extension research by shifting the focus from the mere transference of a specific image and affective response to how the motivation to use primary services shapes the perception of extended services. The findings demonstrate that brand extensions are shaped by existing brand perceptions, as well as the deeper psychological mechanisms driving consumer engagement with the parent brand (Bhaduri, 2019; Gerrath & Biraglia, 2021). This insight broadens the theoretical understanding of brand extensions by introducing trust as a dynamic outcome of consumer motivation rather than a passive transfer of brand associations.

Finally, we contribute to the literature on trust by emphasizing the need to distinguish cognitive and emotional trust in service adoption. Although trust has been recognized as a key determinant of consumer behavior (Chin et al., 2022; Lian & Li, 2021), several studies still treat it as a unidimensional construct (Alalwan et al., 2015; de Blanes Sebastián et al., 2023; Kilani et al., 2023), thus failing to capture its multifaceted nature. Cognitive trust is formed through rational assessments of competence and reliability, whereas emotional trust develops through familiarity and affective connections (Leong et al., 2021; Wang et al., 2023). We highlight that these two trust dimensions are shaped by different consumer motivations; specifically, hedonic (utilitarian) motivation strengthens emotional (cognitive) trust. This distinction is particularly important in service extensions, where rational credibility and emotional comfort influence consumer adoption decisions (Wang et al., 2023). By providing empirical support for a dual-process trust formation mechanism, this study extends the literature on trust and offers a more comprehensive framework for analyzing trust dynamics in extended service contexts.

### 5.2. Practical Implications

This study has several practical implications for marketers across diverse domains, particularly mobile payment marketers, technology product marketers managing service extensions, and general marketers navigating brand expansion. Mobile payment service marketers must cultivate cognitive and emotional trust. Cognitive trust can be reinforced through transparency, security measures, and seamless functionality to ensure that users perceive the payment service as reliable. Emotional trust is built through familiarity, brand consistency, and personalized experiences that create a sense of comfort and engagement. Payment marketers can foster stronger consumer confidence by strategically leveraging both dimensions of trust to encourage wider adoption and sustained usage.

Additionally, tech product marketers designing service extensions should bear in mind that consumer motivation and prior experience with the primary service significantly influence the adoption of extended offerings. Unlike traditional brand extensions that primarily rely on brand image transfer, technology-based service extensions require an understanding of how user engagement with the core platform shapes the perception of new features. This insight has critical implications for the sequence in which technological companies introduce new services. For instance, launching a mobile payment service after establishing a widely used SNS would allow companies to leverage hedonic and utilitarian motivations (Akdim et al., 2022; Anderson et al., 2014; Xu et al., 2012) because users already find value in their social interactions and connectivity (Nan et al., 2020). However, introducing a social networking feature after a payment service is adopted may require a stronger focus on utilitarian benefits because the primary consumer mindset is transactional rather than hedonic. In sum, technology companies should strategically plan the order of service extensions to maximize adoption by aligning them with their user bases’ existing motivations.

Moreover, our findings underscore the broader significance of context in shaping consumer perceptions of the extended products and services. Marketers should consider how brand associations transfer and how consumer experiences and behavioral patterns within the primary service influence expectations for the extension. By acknowledging these contextual dynamics, marketers can develop more effective strategies consistent with consumer expectations and enhance the overall brand extension process.

### 5.3. Limitations and Future Research

First, this study focuses on South Korea, which has a highly developed mobile payment system. Market conditions differ significantly across countries; this affects how trust is formed in digital financial services. For instance, contextual differences—such as varying levels of institutional trust or public attitudes toward new technologies—could influence trust formation, even in technologically advanced environments. Additionally, trust may be shaped by different mechanisms in regions with less mature mobile payment infrastructures. While consumers in mature markets rely on brand reputation, security measures, and technological reliability, those in less-developed markets may place greater trust in community networks, government endorsements, or peer recommendations. Moreover, emotional trust and social proof may play a stronger role in driving adoption where digital financial systems are less developed. Furthermore, the cultural context is another important factor to consider before generalizing our findings. For example, individuals from Eastern societies are often characterized by a holistic processing style (Jang & Kim, 2022) in contrast to the more analytical processing style commonly found in Western societies. Considering these cultural differences in processing style, emotional trust may play a more prominent role in Eastern contexts, whereas cognitive trust may be more influential in Western contexts. Future research could extend the current study by incorporating diverse cultural settings (e.g., Western vs. Eastern societies) to examine how the mechanisms of cognitive and emotional trust may differ across cultures. Such exploration would be particularly relevant for global technology and payment service providers seeking to develop universally applicable user strategies.

Second, we did not consider the extended service’s potential feedback effect on the primary service. For example, the trust gained through the payment service may positively influence the primary service and, thus, reinforce user confidence and engagement. Examining this reverse spillover effect could clarify whether increased trust in the extended service would enhance the trust and usage intention for the core platform, thereby providing deeper insights into the interdependencies within the mobile payment ecosystem. Future research could employ longitudinal or cross-lagged panel designs for a more comprehensive examination of how trust in extended services (e.g., payment) and trust in primary services (e.g., social networking) influence each other over time.

Third, while users’ motivations for using SNSs are likely more diverse than a simple hedonic–utilitarian distinction, our study conceptualized motivation in this binary framework for analytical clarity. Likewise, we simplified the concept of trust into two dimensions—cognitive and affective—though trust may involve more complex and context-dependent components. For instance, our model did not explicitly account for perceived cybersecurity risks, which are typically central to users’ beliefs about the reliability and safety of mobile payment services. Prior studies suggest that perceived risk, particularly concerns regarding privacy and financial security, is a critical antecedent of trust in digital services (e.g., J. Park et al., 2019). While our research focuses on trust perceptions rather than actual risk, we acknowledge that perceived cybersecurity threats significantly influence trust formation and subsequent behavior. Future research should consider individual differences in motivations and perceptions influencing mobile payment adoption. Employing qualitative methods—such as in-depth interviews—may be effective in exploring these psychological factors. Combining qualitative and quantitative approaches would provide a richer understanding of user behavior in integrated social and financial service contexts.

Finally, this study primarily focuses on the relationship between users and the payment service provider; however, trust in e-payment systems is shaped by a wider network of stakeholders, such as financial institutions and merchants. Financial institutions handle transaction processing and authentication, contributing to users’ cognitive trust by ensuring the reliability and security of the payment experience. By contrast, merchants may influence emotional trust more through post-transaction experiences, such as order fulfillment, returns, and dispute resolution. Future research exploring how trust is built and maintained in the broader payment ecosystem could allow for a more comprehensive understanding of trust in integrated digital environments.

### 5.4. Conclusions

With the rapid expansion of mobile payment services, numerous technology companies have integrated such services as extensions of their primary platforms. However, prior research has largely treated these services as independent offerings, thus overlooking how user engagement with the core platform shapes trust in extended financial services. This study addresses this gap by examining how motivations for using an SNS influence trust formation in the payment extension of the SNS, which ultimately shapes behavioral intentions.

By integrating platform-based user motivations with trust-building mechanisms, this study provides a more comprehensive framework for analyzing adoption behavior in technology-driven financial services. The findings offer valuable guidance for technology firms expanding their service ecosystems and emphasize the need to align service extensions with existing user motivations to enhance trust formation, encourage adoption, and strengthen platform engagement.

## Figures and Tables

**Table 1:** Demographic information of participants.

Characteristic	Category	Frequency	Percentage (%)
Gender	Male	243	50.8
	Female	235	49.2
Age (years)	20–29	98	20.5
	30–39	96	20.1
	40–49	84	17.6
	50–59	82	17.2
	60–69	95	19.9
	70–79	23	4.8
Educational level	Below high school	113	23.6
	College/university degree	302	63.2
	Master’s degree	48	10
	Doctorate/Ph.D.	15	3.1
Occupation	Student	43	9
	Housewife	89	18.6
	Company worker	254	53.1
	Self-employed	43	9
	Other occupation	49	10.3

**Table 2:** Reliability and validity of scales.

Construct Items			Factor Loading	AVE	C R	Cronbach’s α
KakaoTalk	Hedonic motivation (HM)	HM1	0.809	0.706	0.944	0.943
		HM2	0.846			
		HM3	0.853			
		HM4	0.873			
		HM5	0.796			
		HM6	0.830			
		HM7	0.872			
	Utilitarian motivation (UM)	UM1	0.745	0.638	0.933	0.933
		UM2	0.716			
		UM3	0.715			
		UM4	0.849			
		UM5	0.873			
		UM6	0.825			
		UM7	0.834			
		UM8	0.814			
KakaoPay	Emotional trust (ET)	ET1	0.840	0.723	0.887	0.886
		ET2	0.841			
		ET3	0.870			
	Cognitive trust (CT)	CT1	0.800	0.720	0.885	0.883
		CT2	0.860			
		CT3	0.883			
	Behavioral intention (BI)	BI1	0.906	0.730	0.843	0.890
		BI2	0.800			

**Table 3:** Test of discriminant validity.

	Mean	SD	HM	UM	ET	CT	BI
HM	4.910	1.197	0.840				
UM	5.768	1.298	0.801 **	0.799			
ET	4.979	1.170	0.647 **	0.622 **	0.856		
CT	5.107	1.182	0.643 **	0.633 **	0.783 **	0.849	
BI	4.779	1.422	0.481 **	0.511 **	0.682 **	0.642 **	0.854

Note: ** *p*-value < 0.01; The diagonal values are the square roots of the constructs’ AVE values.

**Table 4:** SEM results.

Hypothesis		B	S.E.	t-Value	Result
H1	ET--> BI	0.691 ***	0.085	8.108	Supported
H2	CT--> BI	0.299 ***	0.076	3.954	Supported
H3	HM --> ET	0.451 ***	0.077	5.821	Supported
H4	HM --> CT	0.462 ***	0.098	5.667	Supported
H5	UM --> ET	0.297 ***	0.081	3.193	Supported
H6	UM --> CT	0.357 ***	0.093	3.635	Supported

Note: *** *p* < 0.001. B means unstandardized coefficients; S.E. means standard errors.

## Data Availability

The data used in this study are available from the corresponding author upon reasonable request.

## Appendix A. Constructs and Measurement Items

**Constructs**	**Code**	**Items**	**Reference**
Hedonic motivation (HM)	HM1	Using KakaoTalk piques my curiosity.	Salimon et al. (2017)
	HM2	Using KakaoTalk would provide a lot of pleasure.	
	HM3	KakaoTalk has a lot of fun features.	
	HM4	Using KakaoTalk would be always fun.	
	HM5	I am always happy with KakaoTalk’s features.	
	HM6	Using KakaoTalk would make me feel good.	
	HM7	Overall, using KakaoTalk would be enjoyable.	
Utilitarian motivation (UM)	UM1	Using KakaoTalk could provide a lot of information quickly and easily.	Chen et al. (2013); H. Lee and Cho (2020)
	UM2	Using KakaoTalk could provide useful information.	
	UM3	Using KakaoTalk could teach you things you did not know.	
	UM4	Using KakaoTalk could provide helpful information.	
	UM5	Using KakaoTalk is a cheap way to get information.	
	UM6	Using KakaoTalk is helpful to me.	
	UM7	Using KakaoTalk makes my job more productive.	
	UM8	KakaoTalk is a great tool for getting things done quickly.	
Cognitive trust (CT)	CT1	I feel that payments and transfers using KakaoPay are accurate.	Dwyer et al. (2007); G. Kim et al. (2009)
	CT2	I feel that using KakaoPay for payments and transfers is safe.	
	CT3	I feel that using KakaoPay for payments and transfers is reliable.	
Emotional trust (ET)	ET1	I feel secure using KakaoPay services.	Komiak and Benbasat (2006)
	ET2	I feel comfortable using KakaoPay services.	
	ET3	I feel satisfied with KakaoPay services.	
Behavior intention (BI)	BI1	I intend to continue using KakaoPay in the future.	
	BI2	I plan to use KakaoPay more frequently in the future than now.	
